# Structural and optical characteristics determined by the sputtering deposition conditions of oxide thin films

**DOI:** 10.3762/bjnano.12.29

**Published:** 2021-04-19

**Authors:** Petronela Prepelita, Florin Garoi, Valentin Craciun

**Affiliations:** 1 National Institute for Laser, Plasma and Radiation Physics, 409 Atomistilor Street, PO Box MG-36, Magurele 077125, Ilfov, Romania

**Keywords:** magnetron sputtering, optical quality, SiO_2_ and ZnO, structural properties, thin films

## Abstract

The influence of film thickness on the structural and optical properties of silicon dioxide (SiO_2_) and zinc oxide (ZnO) thin films deposited by radio frequency magnetron sputtering on quartz substrates was investigated. The deposition conditions were optimized to achieve stoichiometric thin films. The orientation of crystallites, structure, and composition were investigated by X-ray diffraction (XRD) and X-ray photoelectron spectroscopy (XPS), while the surface topography of the samples was analyzed using scanning electron microscopy (SEM). The optical characteristics were measured for samples with the same composition but obtained with different deposition parameters, such as increasing thickness. The optical constants (i.e., the refractive index *n*, the extinction coefficient *k*, and the absorption coefficient α) of the SiO_2_ and ZnO oxide films were determined from the transmission spectra recorded in the range of 190–2500 nm by using the Swanepoel method, while the energy bandgap was calculated from the absorption spectra. The influence of thickness on the structural and optical properties of the oxide films was investigated. Good optical quality and performance were noticed, which makes these thin films worthy of integration into metamaterial structures.

## Introduction

The application of oxide thin films is quite diverse due to their excellent properties [1–5], such as dielectric properties [6–8] for the production of metamaterials [9]. Metamaterials applied in the field of space science come with a new dimension of microstructural representation of advanced functional materials [10–11]. Metamaterial structures are of significant interest not only in space science but also in the fields of public security and sensors [9–11]. Materials with dielectric properties, such as SiO_2_ and ZnO, are used to build devices with metasurface structures whose properties can be observed in the visible spectrum. They are intensely investigated due to their versatile properties, such as high transmission in the visible range [12–13] and broad energy bandgap [14–16], among others. Among the important applications of these oxides are materials with dielectric properties used in the fabrication of metasurface structures, transparent conductive oxides and buffer layers used in solar cells, and materials used in sensor technology [6,8,17–21]. Materials with dielectric properties (e.g., SiO_2_ and ZnO) exhibit a dependence of the electrical resistance with temperature [22–23].

SiO_2_ and ZnO films are obtained by various deposition techniques, such as matrix-assisted pulsed laser evaporation (MAPLE) [24–25], spin coating of sol–gel precursor solutions [26], radio-frequency magnetron sputtering (rfMS) [27–30], vacuum thermal evaporation (VTE) [31–33], chemical methods [34], reactive ion beam sputter deposition [35], among others. For example, SiO_2_ and ZnO films obtained by rfMS can be either used as dielectric materials in metasurface structures or as dielectric interfaces in the structure of a metamaterial.

This paper reports the experimental conditions for deposition of ZnO and SiO_2_ films as an improvement of the rfMS vacuum deposition technique for dielectric layers (e.g., ZnO and SiO_2_) onto quartz substrates. Here we investigated SiO_2_ and ZnO thin films with thickness values ranging from 200 to 300 nm. Thus, we analyzed the beneficial effect of increasing film thickness on the composition, morphology, structure, and spectral characteristics of the studied samples. This way of analyzing oxide thin film thickness dependence on the optical and structural characteristics allows us to clearly point out the need to use these materials in metamaterial structures.

The novelty of this study is the acquisition of high-performance structural and optical properties of oxide materials deposited by rfMS under optimized conditions. These oxide materials can be, later on, integrated into metamaterial structures to improve their functionality.

## Experimental

The VARIAN ER 3119 EletroRava deposition equipment (available at the National Institute for Laser, Plasma and Radiation Physics, INFLPR) is provided with a deposition chamber, two magnetrons, and in situ thickness monitoring. Thus, rfMS [27,36–37] was used to deposit SiO_2_ and ZnO oxide films. This technique ensures material deposition onto large areas and quality thin films for multiple applications. They are obtained at room temperature on quartz substrates with thickness values ranging from 200 to 300 nm, from targets in the form of a disk with 4″ diameter and 0.125″ thickness. SiO_2_ and ZnO (99.99% purity, Lesker) were individually sintered. Working gases (i.e., argon, 95% and oxygen, 5%) were introduced in the deposition chamber via a circuit provided with flow meters (30 and 1.5 sccm, respectively), in order to precisely control and regulate the flow of gases into the deposition chamber. Quartz (fused silica, NEGS2) slides with dimensions of 2 cm × 2 cm × 0.1 cm were used as substrates. Initially, all substrates were cleaned in an ultrasonic bath to ensure good reproducibility of the properties of the thin films. Subsequently, the substrates were kept in special mounts on a rotating metallic plate, above the deposition targets. The application of the rfMS method results in a uniform growth of the oxide films and a good control of their composition.

To characterize the structure and thickness of the deposited SiO_2_ and ZnO thin films, several methods were applied, including XPS, XRD, and SEM. Hence, the ESCALAB 250+ XPS equipment was used to determine the surface composition of the samples with the following specifications: monochromatic Al Kα radiation (1486.6 eV) and vacuum in the analysis chamber (*p* ≈ 1.6 × 10^−10^ mbar). The XRD analysis was performed using a Brucker D8 Advance diffractometer. The crystalline structure of the oxide thin films was investigated by applying the standard XRD technique using Cu Kα radiation (λ = 1.55418 Å) in the range of 2θ = 25–80 degrees.

By using SEM we studied the surface of the samples at different magnifications (50000× and 20000×) by scanning them with a beam of accelerated electrons at very high energies (≈20 keV). The structural quality and surface morphology were investigated using a scanning electron microscope (FEI Co., model Inspect S50). The system is equipped with an X-ray source and an EDX unit with elementary energy dispersion spectroscopy (EDS). These analyses employ different magnifications depending on the quality of the thin films and the structure of their surface. Using cross-section imaging and a magnification of 20000×, it was possible to gain information related to the thickness of our samples. The high-resolution elementary microanalysis of the cross section perpendicular to the surface of the thin films was performed in manual mode, where the adjustment device allowed the manual setting of the tilt angle from −90° to +90°. Therefore, the dynamic focusing with tilt angle was successfully used. The thickness was measured by the cross-section technique of the SEM analysis, with a margin of error of ±5% (2–5 nm) compared to a standard 100 nm thick sample.

Optical transmission spectra were acquired using a UV–vis–NIR Perkin-Elmer Lambda 950 Spectrophotometer, with a measuring range between 190 and 2500 nm, and a wavelength accuracy of 0.08 nm in UV–vis and 0.3 nm in the NIR band, respectively.

## Results

**Figure 1:**
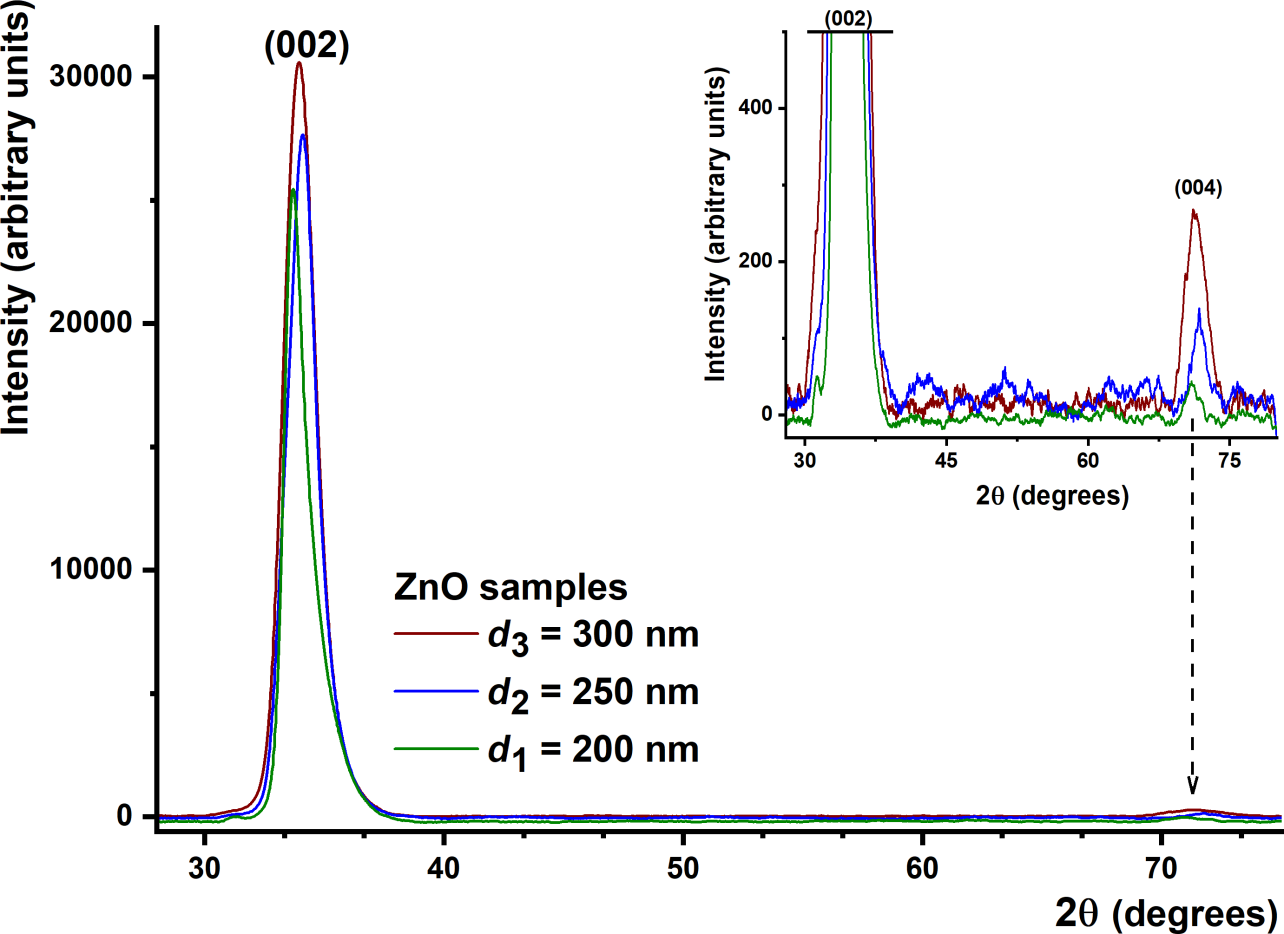
XRD patterns of ZnO thin film samples with different thickness, namely: 200, 250, and 300 nm.

The X-ray diffraction analysis of oxide samples was performed in the angle range of 25–80 degrees (Figure 1). This was done to determine the type of structure (e.g., polycrystalline or amorphous) and orientation of the thin films. Figure 1 shows typical XRD patterns of ZnO thin films with increasing thickness and prepared via rfMS. Following the effect of the deposition parameters of the oxide films we found that the diffractograms show an increase of peak intensity with thickness, which determines an improvement of their crystalline structure. In the case of ZnO samples, the crystalline phases and peaks were identified as corresponding to (002) and (004) planes, according to JCPDS XRD, the standard diffractograms [38]. These diffractograms indicate good crystalline quality and the analyzed films show two diffraction peaks, characteristic of the ZnO hexagonal structure (wurtzite). The studied thin films have a crystalline structure with a strong orientation of the planes (002) parallel to the surface of the substrates.

In the diffractogram of the 200 nm thick film, diffraction peaks corresponding to a single-phase growth of the film were initially identified. Low intensities of the diffraction lines (004) are due to growth stress, which is unevenly distributed in the film. In the diffractogram corresponding to the 250 nm thick film it can be seen how the film grew oriented with the *c*-axis perpendicular to the substrate surface, a phenomenon that is specific to depositions made at room temperature.

The values of the (002) plane corresponding to the multiple reflections on the substrate surface for ZnO thin films are presented in Table 1. One of the films with the highest quality in terms of structure was the one deposited at a thickness of 300 nm. This is consistent with the value of the lattice parameter *c* = 5.2090 Å, indicating a good oxygenation.

**Table 1:** XRD results for ZnO thin films with different thickness values.

sample	*d* (nm)	(hkl)	2θ (°)	β_2θ_ (mrad)	*D* (nm)	*d*_hkl_ (Å)	*c* (Å)	ε2 (%)	TC (hkl)

ZnO200	200	(002)	34.42	63.0	23.0	2.604	5.209	0.088	1.82
		(004)	72.57	49.7	34.5	1.302	5.208	0.069	1.05
ZnO250	250	(002)	34.43	50.9	28.4	2.604	5.208	0.067	1.98
		(004)	72.58	44.1	38.9	1.301	5.207	0.057	1.88
ZnO300	300	(002)	34.42	41.3	35.1	2.604	5.209	0.078	2.19
		(004)	72.58	39.1	44.0	1.302	5.208	0.061	3.55

*d* – thin film thickness; (hkl) – Miller indices corresponding to diffraction planes; θ – Bragg angle; β – half width of the diffraction peak; *D* – size of the crystallites [39]; *d*_hkl_ – interplanar spacing, *c* – crystal lattice constant, with λ = 1.54 Å, wavelength of the incident radiation, and ε2 – tension values along the *c*-axis [40]; TC (hkl) – texture coefficient of the (hkl) plane [41–42].

The 300 nm thick ZnO film proved to be improved in structural performance as compared to the other films obtained under similar deposition conditions. This conclusion is supported by the increase in size of the ZnO crystallites and values of the texturing coefficient, which are greater than 1, showing that a larger number of crystallites are oriented with the (hkl) planes parallel to the substrate surface. The narrower the diffraction peaks, the larger the crystallite size. The lattice parameters of the deposited films have lower values than the standard ones [40]. This can be attributed to the fact that the films are made without being subjected to a thermal treatment, which induces an internal stress in the ZnO thin films. To the same extent, with the depositions made at room temperature on all SiO_2_ thin films, their structure proved to be essentially amorphous [28,37] with no sharp XRD reflection lines and featuring a matte surface. Based on XPS measurements the effect of thickness increase on the deposited samples and the oxidation states of each element were evaluated. The properties of the SiO_2_ thin films were also determined, noting the preservation of the stoichiometry and purity of the preparation technique.

The XPS method characterizes the first 2 to 6 nanometers of the surface of a sample, including the contaminant layer. The chemical composition of oxide films, obtained by XPS, can be slightly different from that of the target due to surface diffusion phenomena and surface-specific chemical processes in contact with the atmosphere. The XPS measurements of the analyzed samples were performed with photons that cause no major disturbances on the bombarded surface.

**Figure 2:**
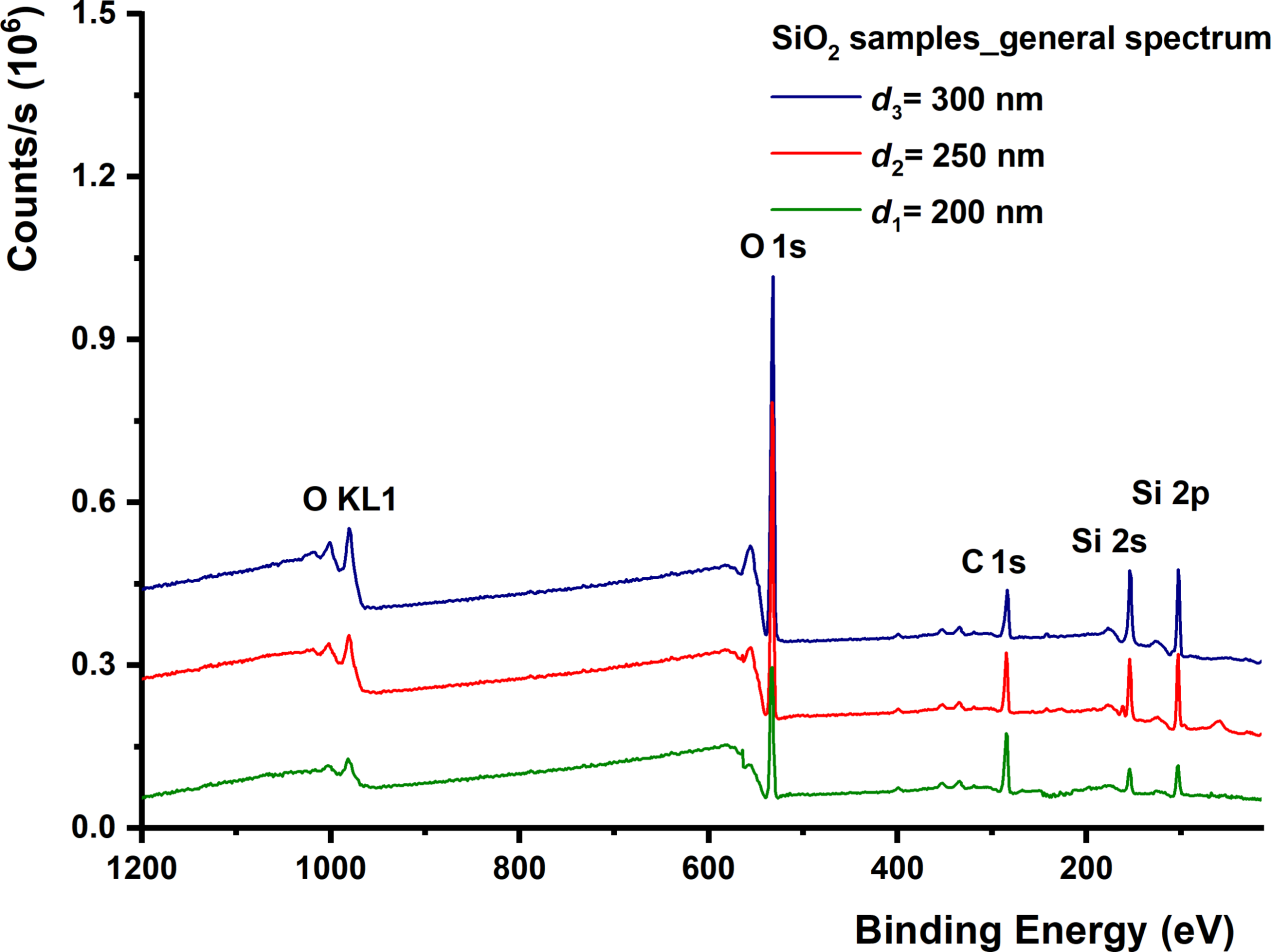
XPS patterns of SiO_2_ thin films: general spectra.

To keep the contamination layer from the surface of the films, they were not sputtered using the ion gun. This is because sometimes the sputtering affects the stoichiometry of the samples by depleting the films of oxygen. Figure 2 shows the general oxide spectra for three SiO_2_ samples.

**Figure 3:**
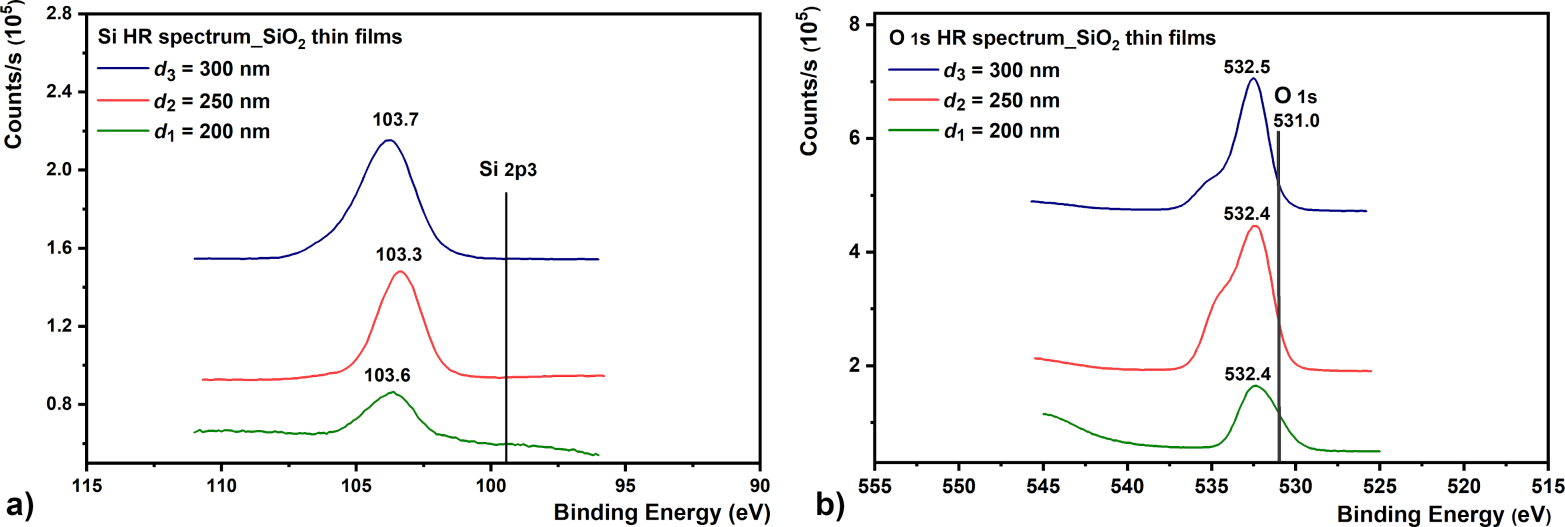
High-resolution XPS spectra acquired from SiO_2_ samples: (a) Si 2p3 and (b) O 1s.

The high-resolution (HR) analysis of the Si 2p3 and O 1s spectra recorded [43–44] for the SiO_2_ samples are shown in Figure 3. Using this analysis, we determined the elemental composition as well as the chemical and electronic states of the elements that exist in the SiO_2_ films. Although Si 2p3 shows small chemical changes, the binding energy value of 103.7 eV indicates a completely oxidized Si for the SiO_2_ films (Figure 3a) [43–46]. Experimental data reveal that there is good stoichiometry for this film.

**Figure 4:**
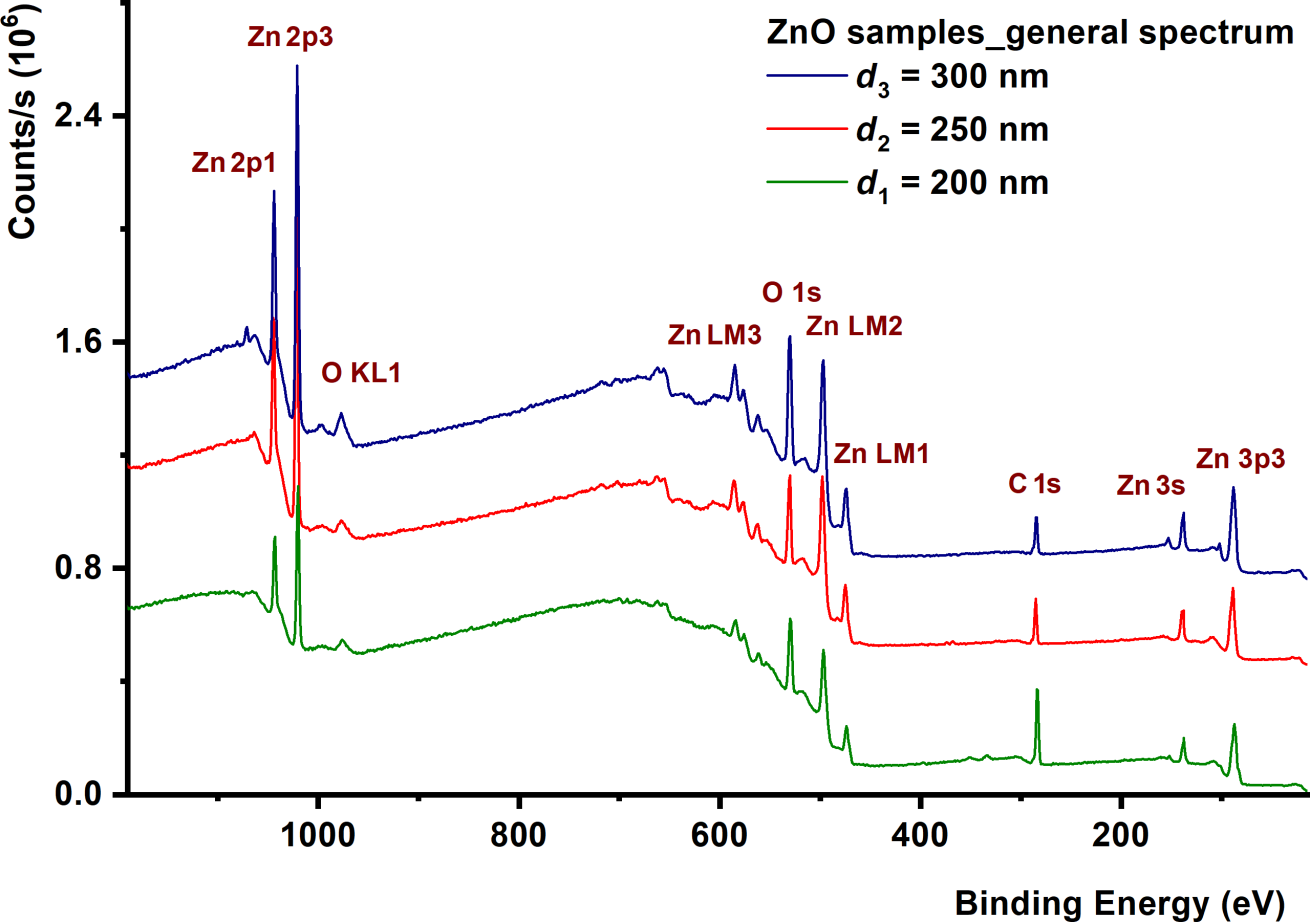
XPS survey spectra acquired from ZnO films.

The general spectra of ZnO samples are shown in Figure 4, where peaks corresponding to C 1s, O 1s, and Zn 2p were identified for all investigated samples. The presence of photoelectron signals, ZnO electrons, and carbon contamination at the binding energy value of 284.8 eV in the inherently contaminated film are observed.

The carbon peak represents the carbon absorbed at the surface, after which the energy calibration of the other spectra was done [47–48]. The contaminated carbon layer in the atmosphere becomes increasingly thinner with increasing film thickness. This is a possible explanation for the decrease in carbon peaks as the thickness of the oxide films increases. It must be said that no metallic elements are identified; however, they are in oxide bonds just like in oxide compounds.

**Figure 5:**
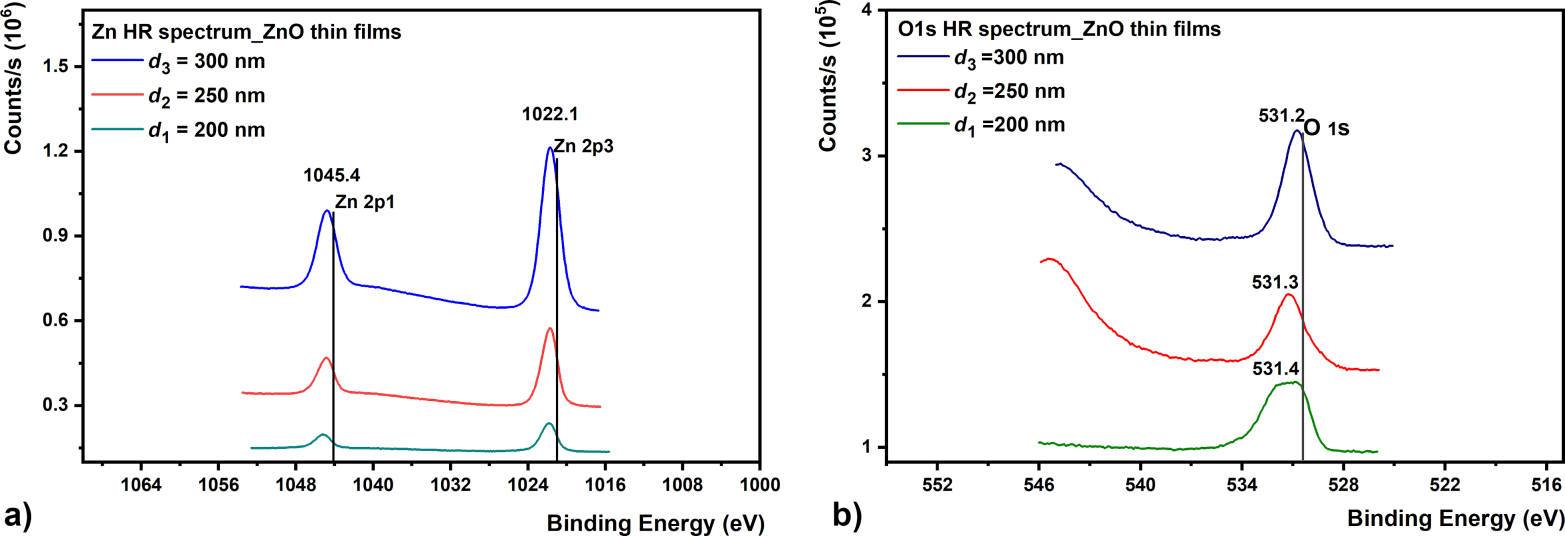
High-resolution (a) Zn 2p3 and (b) O 1s XPS spectra acquired from ZnO samples.

This carbon contamination obviously decreases as the film thickness increases. An increase in Zn 2p and O 1s intensities is also observed [49–50]. Figure 5 shows the HR spectra of the electron distribution at Zn 2p and O 1s levels recorded for the ZnO samples.

**Figure 6:**
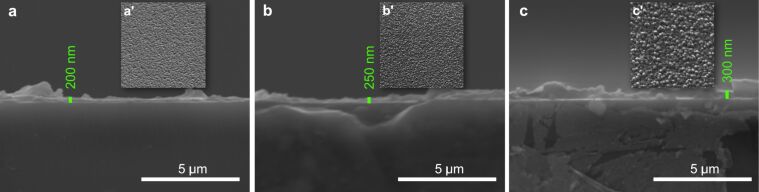
Cross-sectional SEM images of the cleaved SiO_2_ films with measured thickness values of (a) *d*_SiO2_ = 200 nm, (b) *d*_SiO2_ = 250.3 nm, and (c) *d*_SiO2_ = 300 nm. SEM micrographs of the surface morphology for the corresponding thin films are also depicted: (a’) *d*_SiO2_ = 200 nm, (b’) *d*_SiO2_ = 250 nm, and c’) *d*_SiO2_ = 300 nm.

**Figure 7:**
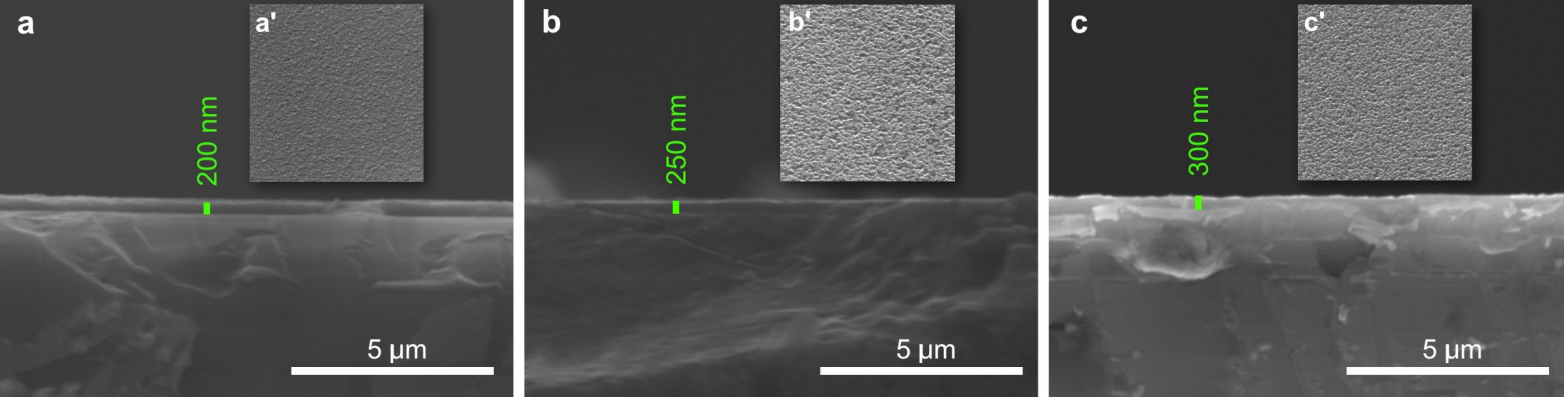
Cross-sectional SEM images of the cleaved ZnO films with measured thickness values of (a) *d*_ZnO_ = 200 nm, (b) *d*_ZnO_ = 250 nm, and (c) *d*_ZnO_ = 300.2 nm. SEM micrographs of the surface morphology for the corresponding thin films are also depicted: (a’) *d*_ZnO_ = 200 nm, (b’) *d*_ZnO_ = 250 nm, and (c’) *d*_ZnO_ = 300 nm.

As we can see in Figure 5a, the Zn 2p spectrum contains a doublet with binding energy values of 1022.1 and 1045.4 eV, which correspond to the Zn 2p3 and Zn 2p1 lines, respectively [47]. The presence of metallic zinc is not observed in the general spectrum, which indicates that we have only oxidized zinc in the film. These results are in good agreement with those in the literature [49–51]. Using a magnification of 20000×, the SEM analyses show small structural changes in the quality of the SiO_2_ and ZnO films, which are proportional to their increase in thickness. SEM cross-section images (Figure 6) show films with a dense, homogeneous, and uniform surface. The structural analysis of the surface of SiO_2_ (Figure 6a’–c’) and ZnO (Figure 7a’–c’) thin films is also provided by SEM surface measurements. The surfaces are smooth, with small differences between the surface morphology of a thinner oxide film as compared to a thicker one.

For ZnO samples, there is a tendency for self-structuring of the deposited films, as depicted in the SEM images (Figure 7a–c).

The morphology of these samples was verified using the depth penetration technique of SEM, where it was evidenced that the analyzed films were compact. We could also observe an increase in granulation of the samples with an increase in thickness.

The measured thickness values for the oxide films are shown in Table 2 and they are found to be similar to the predefined ones. The EDS distribution in all investigated samples showed the presence of chemical elements such as Zn L, Si K, and O K (Table 2).

**Table 2:** Typical chemical composition of SiO_2_ and ZnO thin films determined by EDS measurements.

Sample	*d* (nm)	Element	Weight %	Atomic %

SiO_2_ 200	200	O K	35.36	48.38
		Si K	64.64	51.62
SiO_2_ 250	250	O K	47.11	60.64
		Si K	52.89	39.36
SiO_2_ 300	300	O K	50.50	65.88
		Si K	49.50	34.11
ZnO 200	200	O K	17.56	45.73
		Zn L	82.44	54.27
ZnO 250	250	O K	18.14	47.52
		Zn L	81.86	52.48
ZnO 300	300	O K	25.85	49.38
		Zn L	74.15	50.62

*d* – thin film thickness.

In the case of the 200 nm thick SiO_2_ sample, the percentage composition allowed us to observe the stoichiometry of the sample and a depletion of oxygen (48.38%) in the oxide film. For the 250 nm thick SiO_2_ sample, and given the percentage data, following carbon contamination (according to the XPS results) the amount of O K increases (as compared to the 200 nm thick sample), while the amount of Si K decreases. The atomic concentration of silicon decreases very slightly from 51.62% to 34.11%. The EDS analysis of the surface of the SiO_2_ oxide films illustrates an increase in the amount of oxygen as the film thickness increases. In addition, the mass concentration of silicon decreases from 64.64% to 49.50%, accompanied by an increase in the oxide content from 35.36% to 50.50%. The same target was used, with a standard chemical composition and good stoichiometry. The deposited thin films have a chemical composition with values close to this target, especially for thicker samples. For ZnO films an increase in the film thickness resulted in an increase in the atomic oxide (from 45.73% to 49.38 %), as a result of plasma chemical processes.

**Figure 8:**
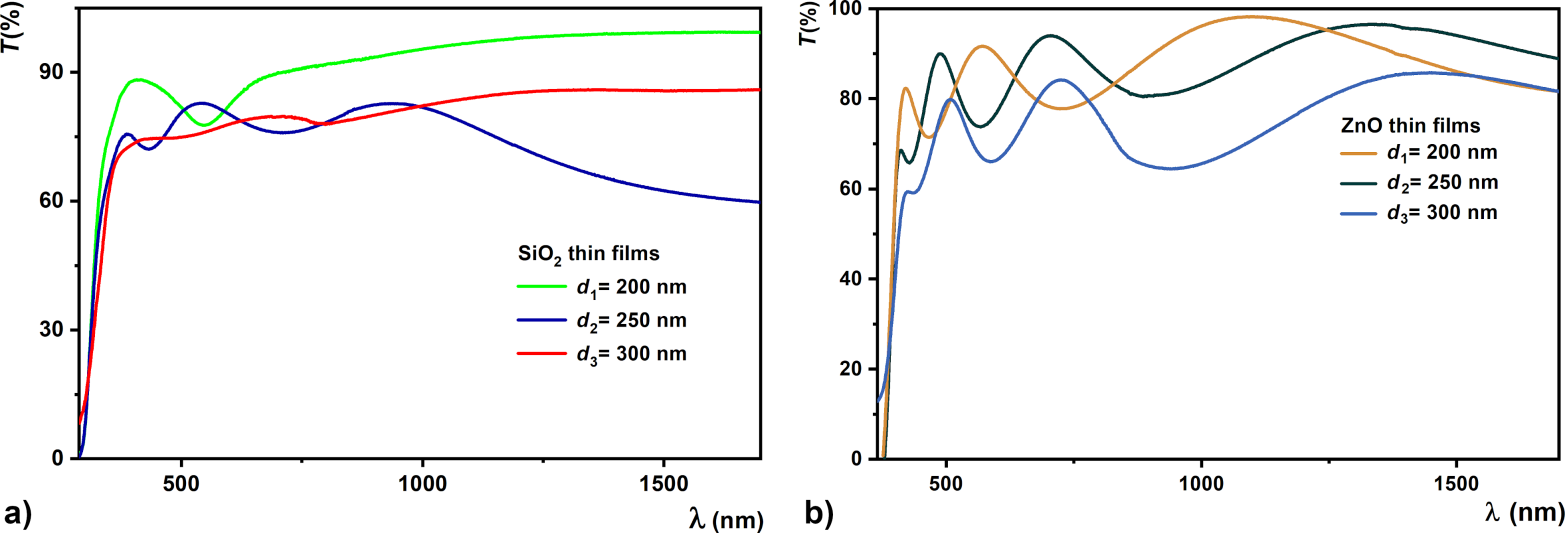
Transmission spectra of thin films with various thickness values (200, 250, and 300 nm): (a) SiO_2_ and (b) ZnO.

The analysis of optical properties, by the absorption of light in the dielectric films of ZnO and SiO_2_ with various thickness values, and the determination of the optical constants are useful for their integration into the design and construction of metamaterial structures. The optical properties of the films were characterized based on the transmission spectra (Figure 8) and the Swanepoel model was chosen to determine the optical constants of the oxide films in the spectral range of 190–2500 nm. The wavelength dependence of the transmission coefficient is depicted for the set of SiO_2_ and ZnO samples with different thickness values.

The presence of maxima and minima in the transmission spectra of SiO_2_ and ZnO thin films allows for the determination of their optical constants with the help of the envelope method proposed by Swanepoel [52–53]. It was found that thinner samples have a transmission in the range of 78–92% while thicker ones have a transmission in the range of 82–65%, for radiation with a wavelength of 600 nm.

The interference maxima and minima (*T*_M_ and *T*_m_, respectively) are located on two curves of the transmission spectra (Figure 8). They are called envelopes and defined by the continuous functions:

[1] $$T_{\text{M}}=\frac{16n^{2}n_{S}e^{-\alpha d}}{{\left(n+1\right)}^{3}(n+n_{S}{}^{2})-2(n^{2}-1)(n^{2}-n_{S}^{2})e^{-\alpha d}+{\left(n-1\right)}^{3}(n-n_{S}^{2})e^{-2\alpha d}}$$

[2] $$T_{\text{m}}=\frac{16n^{2}n_{S}e^{-\alpha d}}{{\left(n+1\right)}^{3}(n+n_{S}{}^{2})+2(n^{2}-1)(n^{2}-n_{S}^{2})e^{-\alpha d}+{\left(n-1\right)}^{3}(n-n_{S}^{2})e^{-2\alpha d}}.$$

In the case of medium and low absorption domains we obtained [52–53]:

[3] $$\frac{1}{T_{\text{m}}}-\frac{1}{T_{\text{M}}}=\frac{\left(n^{2}-1)(n^{2}-n_{S}^{2}\right)}{4n^{2}n_{S}}.$$

From the above relationship, we can write:

[4] $$N=2n_{S}\frac{T_{\text{M}}-T_{\text{m}}}{T_{\text{M}}T_{\text{m}}}+\frac{n_{S}^{2}+1}{2}$$

and the refractive index becomes:

[5] $$n={\left[N+{\left(N^{2}-n_{S}^{2}\right)}^{1/2}\right]}^{1/2}.$$

The stronger absorption of the 300 nm thick thin films is influenced by the increase in the volume of intercrystalline regions [47,54].

**Figure 9:**
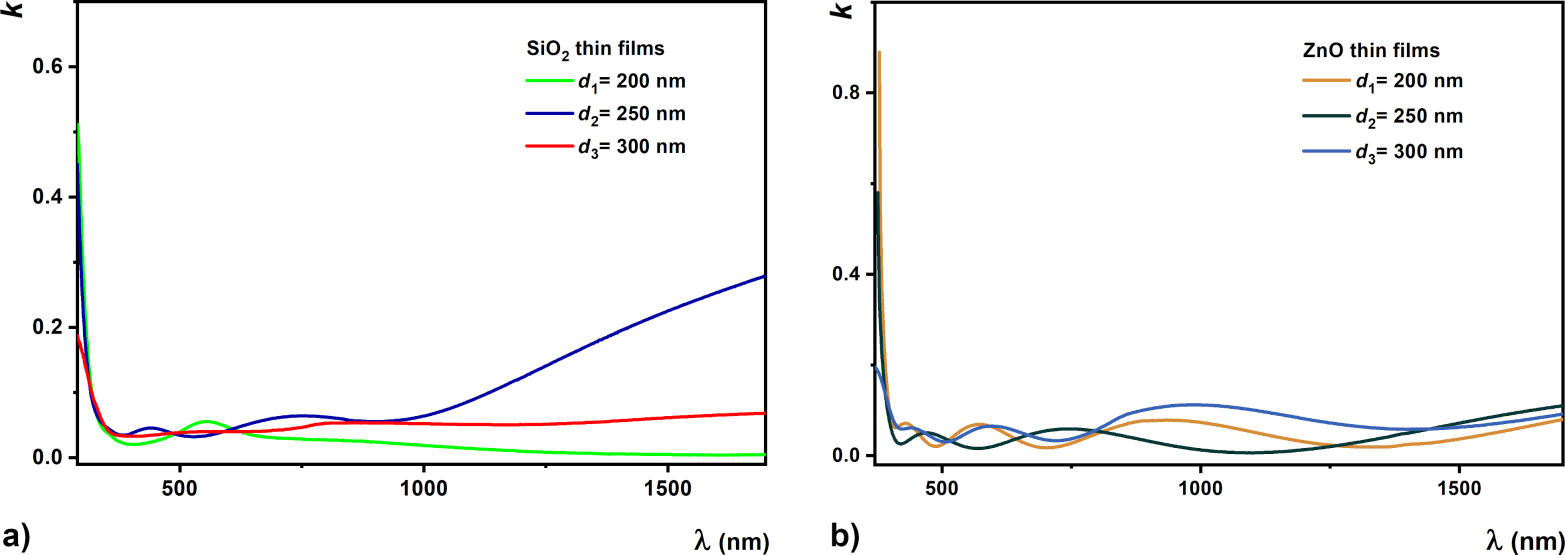
Extinction coefficient dependence on the wavelength for (a) SiO_2_ and (b) ZnO thin films.

Optical constants were calculated for both high and low absorption ranges. In the case of transparent SiO_2_ and ZnO thin films, between the extinction coefficient, *k*, (Figure 9) and the absorption coefficient, α, there is this following relation:

[6] $$\alpha =\frac{4\pi k}{\lambda },$$

where λ is the wavelength.

We determined the optical bandgap, corresponding to the direct optical transitions, by extrapolating the linear portion of the dependency (α*h*ν)^2^ = *f*(*h*ν) to (α*h*ν)^2^ → 0.

**Figure 10:**
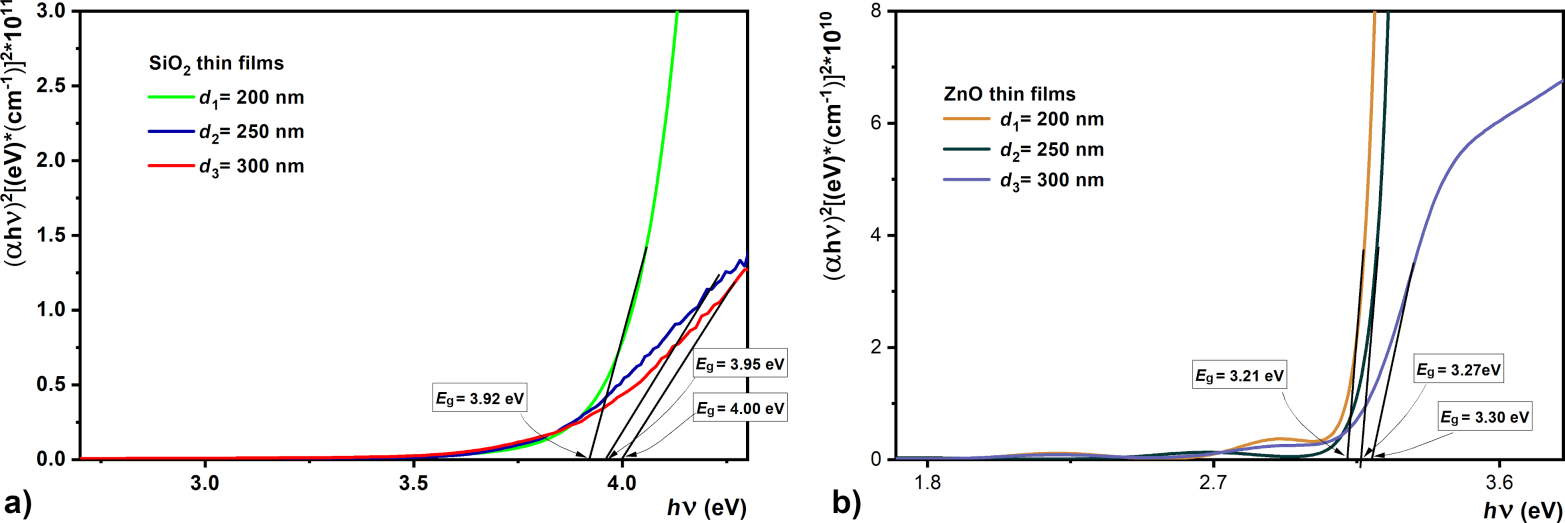
Dependence of (α*h*ν)^2^ = *f*(*h*ν) on the energy of the incident photons for (a) SiO_2_ and (b) ZnO samples with different thickness values.

The edge of the absorption band shifts to longer wavelengths with an increase in thickness of the thin films (Figure 10). In such dielectric materials the electrons are characterized by a high-energy bandgap [55].

The bandgap value was determined for all sets of samples. The obtained values were between 3.92–4.0 eV for the SiO_2_ samples and between 3.2–3.3 eV for ZnO films. The band difference of the ZnO films indicated a direct band-to-band transition between the valence and conduction band, while the film stress determined the improvement of the bandgap.

The obtained bandgap values corresponding to direct transitions are similar to those obtained by other researchers [56–57]. These optical properties of ZnO and SiO_2_ films have proven to be very important for their use in metamaterial structures. With an increase in thickness of the SiO_2_ and ZnO films, the transmission in the visible range decreases and the porosity at the film surface increases, which is justified by the lack of applied heat treatment.

**Figure 11:**
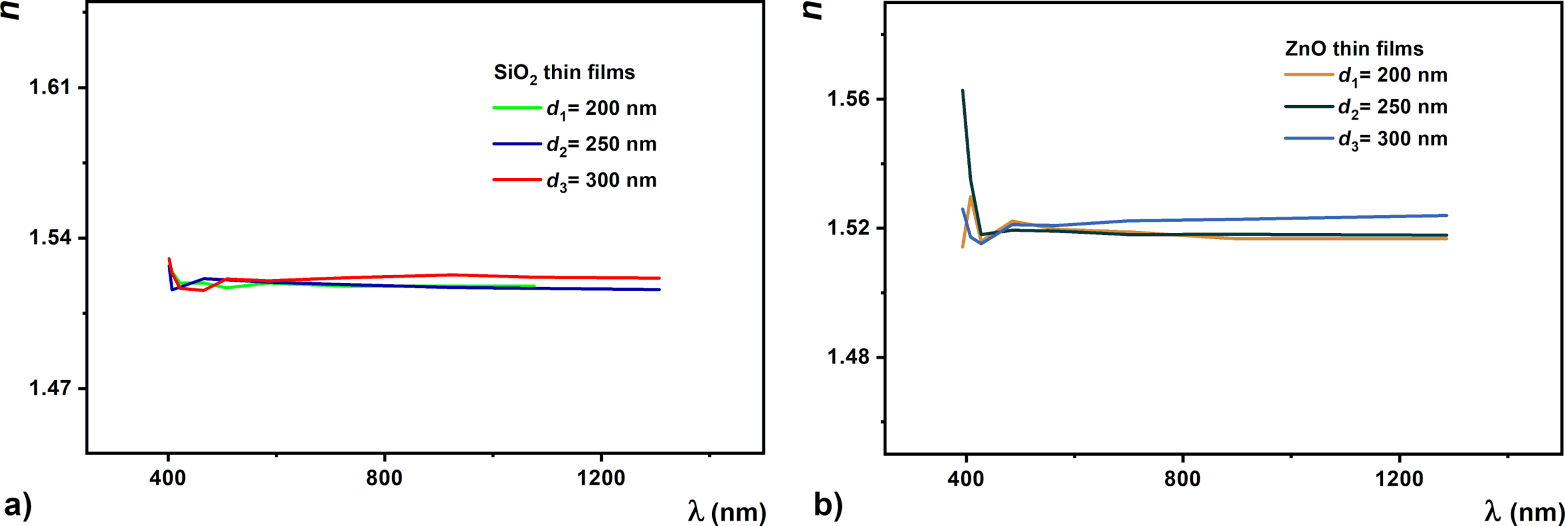
Refractive index dependence on the wavelength (dispersion) for (a) SiO_2_ and (b) ZnO thin films.

The dispersion of the refractive index for the investigated samples shows a normal dispersion in the considered spectral range (Figure 11). The plots indicate that at a wavelength of 430 nm the refractive index of the films approaches a minimum value. The quality of the dielectric materials is also determined by the dielectric constant values.

The values of the dielectric constant were obtained by using the Drude method [58–59] and the spectral absorption of the oxide films. This allowed for the assessment of the permittivity and polarizability of the material, as well as the density of states in the band interval. Based on calculus, the value of the real dielectric constant (ε_r_) can be obtained by:

[7] $$\varepsilon_{\text{r}}=n_{2}-k_{2},$$

and the relationship to compute the imaginary dielectric constant is:

[8] $$\varepsilon_{\text{i}}=2nk,$$

where *n* is the refractive index and *k* is the extinction coefficient.

**Figure 12:**
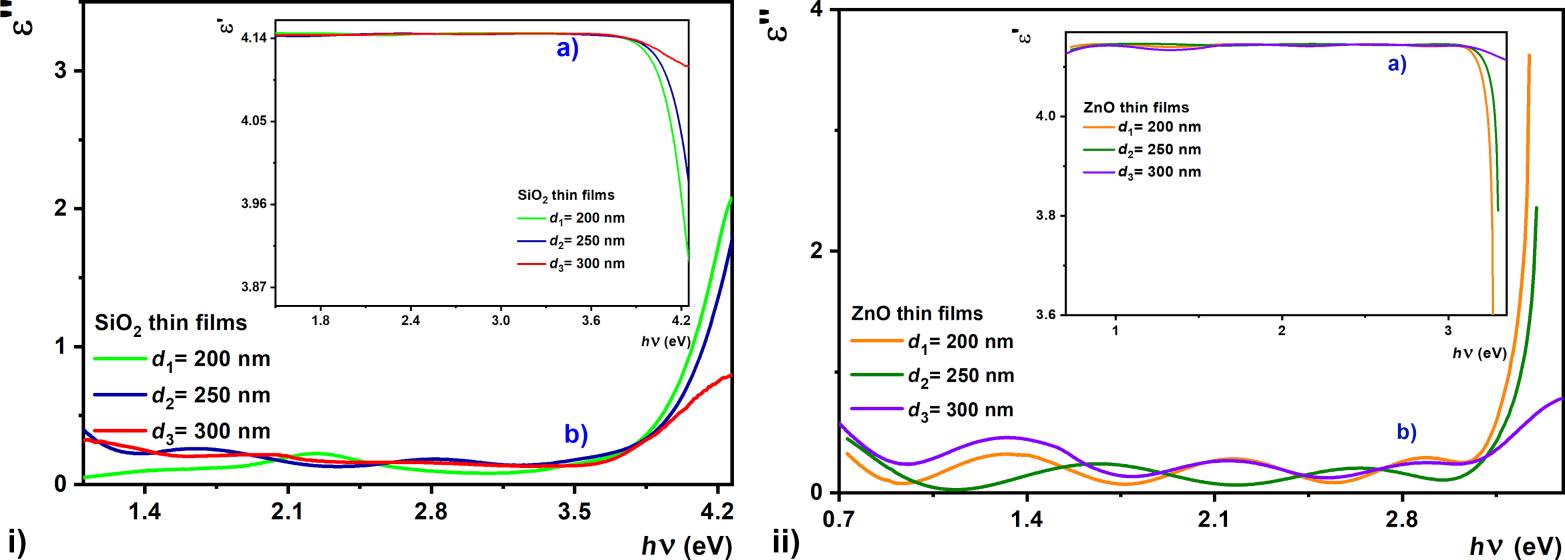
Photon energy dependence of the (a) real and (b) imaginary parts of permittivity for SiO_2_ (i) and ZnO (ii) thin films, with different thickness.

Figure 12 shows the variations of real and imaginary parts of the dielectric constants of ZnO and SiO_2_ films with photon energy. It can be seen that the value of the real part is higher than that of the imaginary part, which is more evident in the 300 nm thick sample. The attractive characteristics of the thickest sample (i.e., 300 nm) suggest that optimal deposition conditions have been obtained for good performance, and there is a possibility to integrate those into metamaterial structures.

Recent studies highlight the innovative applications of ZnO due to its dielectric features (Figure 12) in the form of thin films, but also as thin layers of ZnO nanoparticles synthesized in the gaseous phase as gate dielectrics. Our results show a similar value when compared, for example, with the properties obtained for the SiO_2_–ZnO mixture in different proportions of weight [60].

Thus, by increasing the thickness of ZnO and SiO_2_ films by 100 nm (from 200 to 300 nm) the quality of the samples is improved by 12–15%, resulting in good stoichiometry, increased crystallinity, and improved optical and dielectric properties.

Transparent oxides are an attractive class of plasmonic materials which are under intense study for their integration into low-loss metamaterial structures and a series of applications in transformation optics, sensors, and imaging. Here we used oxide thin films and studied their optical properties (Figure 9 and Figure 11) to be integrated in metamaterials with low refractive index. The results show that a slight variation of the permittivity of the dielectric material (Figure 12) due to the redistribution of electrons is negligible in all cases. The importance of these transparent dielectric oxide films (SiO_2_ and ZnO) in the structures of metamaterials, such as electrically adjustable or low-loss metamaterials, consists in the fact that they improve their quality through their good properties [61–63]. In particular, these thin films were selected for their properties related to a high dielectric constant and low conductivity.

Metamaterial structures with similar repetitive geometric structures, such as Si/SiO_2_/ITO/Au [62], were obtained worldwide. In the case of our research, the new suggested metamaterial structure will consist of dielectric (SiO_2_)/inductive elements/dielectric (ZnO). In the future, these oxide materials can be integrated into metamaterial structures for space microsatellites, for example.

## Conclusion

SiO_2_ and ZnO oxide thin films of various thickness values were deposited by using radio frequency magnetron sputtering. Films with a good stoichiometry were obtained from a source target. In the case of SiO_2_ thin films, it was confirmed by X-ray diffraction measurements that all structures were amorphous.

Following X-ray diffraction analyses, it was proved that ZnO films show an orientation with the *c*-axis perpendicular to the substrate surface. The results indicate that ZnO thin films are crystalline with a hexagonal structure. By increasing film thickness, the crystallinity of the films as well as the size of the crystallites for the (002) plane increase.

The transmission spectra of the studied oxide films are strongly influenced by the deposition conditions. Smaller values for the transmission coefficient were obtained in the case of thicker samples (i.e., 74% for ZnO 300 and 68% for SiO_2_ 300, respectively). Also, an improvement of the optical properties of the thin films with increasing thickness was noticed. In conclusion, some of the best quality SiO_2_ and ZnO films in terms of structure and optical properties were the 300 nm thick ones. Thus, the attractive characteristics of the thickest sample suggest that optimal deposition conditions have been found, allowing us to obtain samples with a good performance to be integrated into metamaterial structures.
